# Description of *Ficus carica* L. Italian Cultivars II: Genetic and Chemical Analysis

**DOI:** 10.3390/plants14081238

**Published:** 2025-04-18

**Authors:** Raffaella Petruccelli, Cristiana Giordano, Deborah Beghè, Lorenzo Arcidiaco, Alessandra Bonetti, Francesca Ieri, Margherita Rodolfi, Tommaso Ganino

**Affiliations:** 1 Institute of BioEconomy, CNR, Via Madonna del Piano 10, Sesto Fiorentino, 50019 Florence, Italy; raffaella.petruccelli@cnr.it (R.P.); cristiana.giordano@cnr.it (C.G.); lorenzo.arcidiaco@cnr.it (L.A.); 2 Economics and Management Department, University of Parma, Via J.F. Kennedy 6, 43125 Parma, Italy; 3 Research Institute on Terrestrial Ecosystems, National Research Council (CNR-IRET), Via Madonna del Piano n. 10, Sesto Fiorentino, 50019 Florence, Italy; 4 Institute of Biosciences and Bioresources (CNR-IBBR), Via Madonna del Piano n. 10, Sesto Fiorentino, 50019 Florence, Italy; francesca.ieri@ibbr.cnr.it; 5 Food and Drug Department, University of Parma, Parco Area Delle Scienze, 27/a, 43124 Parma, Italy; margherita.rodolfi@unipr.it (M.R.); tommaso.ganino@unipr.it (T.G.)

**Keywords:** fig tree, SSRs, polyphenols composition, antioxidant activity, sugars, fig biodiversity

## Abstract

*Ficus carica* L., present in Italy since ancient times, is represented by numerous cultivars grown in both southern and central regions. In recent years, local accessions, as a source of genetic biodiversity, have gained increasing interest for both genetic conservation and their agronomic characteristics, which are well suited for ‘sustainable agriculture’. Based on these considerations, we conducted a parallel study combining the genetic analysis (SSRs marker) and chemical profiling (polyphenols and antioxidant capacity) of fig leaves to characterize 15 cultivars of the Tuscany region. A genetic analysis performed using seven SSR oligonucleotide primers successfully allowed the discrimination of the cultivars studied, with primers MFC2, MFC3, and LMFC30 exhibiting the highest polymorphism. The phytochemical profiling of the leaves, conducted by HPLC-DAD-TOF-MS, revealed 17 phenolic compounds, among which caffeic acid derivatives were the most abundant. The psolaren compound was absent only in Gigante di Carmignano cv. The antiradical capacity varied among cultivars, with Perticone, Brogiotto Nero, and Paradiso exhibiting the highest antiradical capacity. Additionally, Brogiotto Bianco, Corbo, Dottato, Paradiso, Pecciolo Nero, and Verdino contained the highest concentrations of total sugars. Statistically significant differences were observed in total polyphenol content (values ranging from 14.1 to 36.6 mgGAE/gDW) and in flavonoid content (ranging from 25.7 to 52.3 mgQE/gDW). In terms of sugars, the sum of fructose, glucose, and sucrose ranged from 20.60 (Bianco di Carmignano) to 49.24 mg g^−1^ (Verdino), with fructose present in the highest amounts. In conclusion, the 15 cultivars were completely characterized genetically and chemically, offering valuable insights for both conservation strategies and agricultural applications.

## 1. Introduction

Mediterranean fig (*Ficus carica* L.) also known as “common fig”, “edible fig”, or “cultivated fig” [1], is a member of the mulberry family (Moraceae). This species is one of the most representative fruit trees that exemplifies long-term human–tree coexistence, leading to its domestication and/or pre-cultivation management and dispersal beyond its original distribution [2]. The cultivated fig is widespread from the Mediterranean area, with cool winters and hot, dry summers, to tropical and subtropical regions where it is found in natural habitats as a landscape element or cultivated for commercial purposes [3]. It is undoubtedly a traditional crop in many Mediterranean countries, where more than 90% of the cultivated area and over 80% of fig production is concentrated. Among fruit trees, the fig is recognized as an extremely polymorphic species with a rich variety of diversity. This wide variation is due to several characteristics, such as longevity and rustic habits and the ease of cultivation and vegetative propagation, which have led to the spread of many wild forms or landraces. As a result, adaptation to different soil and climatic conditions has favored the emergence of new genotypes and local populations that vary morphologically, molecularly, and agronomically. *Ficus carica* is characterized by a high number of cultivars, and many authors have described fig cultivars [4,5]. To date, the number of cultivars described ranges from 600 to 800, although this number might not include local genotypes that have never been documented.

In recent years, fig has been gaining increasing attention for the fruit’s nutritional properties and exceptional resilience to climate change, thriving in scorching climates with low water availability. The fig is a natural source of minerals, sugars, organic acids, and bioactive compounds beneficial to human health. Numerous bioactive substances, such as phenolic compounds, anthocyanin structures, triterpenoids, coumarins, phytosterols, organic acids, and volatile composites, were identified in the leaves, bark, peels, and flesh of fig cultivars. These biomolecules present a wide range of biological properties with health-promoting functions (e.g., antidiabetic, antipyretic, antioxidant, and antilipemic activities; anticancer bioactivities) able to fight some degenerative diseases [6,7,8]. The diversity of these metabolites is mainly valued as a source of products with potential impacts on human health, but it also plays an important role in defining the fig’s overall phenotype. For this reason, numerous programs have been initiated across Mediterranean countries to characterize fig cultivars and ensure their proper conservation. As part of these efforts, the field of biodiversity collections has been established in various Mediterranean nations, including Italy, Turkey, France, and Spain [9]. Over time, in order to improve the fig genetic collections, new genotypes and accessions have been characterized from morphological, chemical, biochemical, and genetic perspectives. Phenotypic characterization is a traditional approach to discriminate fig cultivars. Many studies have extensively characterized morphological, phenological, and pomological traits (including fruit quality) in combination with multivariate statistical approaches to assess genetic variation and relationships between fig genotypes and cultivars in different countries, such as Italy [10,11], Turkey [12,13], Tunisia [14,15], and Spain [16,17,18]. Although analyzing morphological traits can lead to confusion or misidentification due to the species’ distinctive characteristics and phenotypic plasticity, morpho-agronomical evaluation remains an essential first step in the characterization of fig cultivars. In recent decades, molecular methods, especially DNA-based fingerprinting techniques, have flanked morphological characters, and several studies have been undertaken in this direction using morphological as well as molecular and genetic diversity as well as genotype identity. Plant characterization has been revolutionized by PCR-DNA technologies, and several studies have been reported on the identification of fig cultivars using different molecular markers such as RAPD [19,20,21], ISSR [22], and iPBS retrotransposon [23]. Currently, microsatellites or simple sequence repeats (SSRs) have become the genetic markers of choice for assessing the genetic diversity of the fig trees. Studies on the many fig cultivars or germplasm collections of different Mediterranean countries such as Greece [24,25], Turkey [26,27], Tunisia [28,29], Algeria [30], Italy [31,32], and Spain [17,33] have been published.

Morphological and genetic variability is also reflected in biochemical profiles; consequently, a wide range of compounds can serve as chemotaxonomic markers [34,35]. Plant secondary metabolites, often known as phytochemicals, are non-nutritive plant metabolites that are crucial for plant survival, healthy growth, and reproduction. These compounds are synthesized in order to defend and protect the plants against biotic or abiotic stresses [36]. Most of these components have biological properties that regulate animal biochemistry and metabolism and have the potential to impact human health. In recent years, plant secondary metabolites have played an important role, not only for their pharmaceutical importance but also in taxonomy [37]. Chemotaxonomy supports putative classifications inferred from other characteristics, such as morphology, which is often not sufficient to satisfactorily classify living organisms [38]. Secondary metabolites are often differentially distributed among limited taxonomic groups, so they can provide a metabolite diversity map useful for solving taxonomic problems and building phylogenetic relationships [39,40].

The aim of this study was to describe Italian fig cultivars through the genetic (SSR markers) and chemical characterization of fig leaves. This research is the conclusion of previous work focused on the morphological characterization of the same 15 cultivars, where wide variability among entities has already been shown [41].

## 2. Results and Discussion

### 2.1. Genetic Analysis

In this study, 15 fig cultivars (as listed in the Materials and Methods Section) were analyzed. For genetic characterization, we used seven oligonucleotide primers for simple sequence repeat (SSR) analysis, which was carefully selected by the MFC [42] and LMFC [33] series of microsatellites, formerly developed and successfully employed in fig. All the markers produced polymorphic and reproducible amplification fragments, allowing for the discrimination of the studied fig accessions and identifying a total of 38 alleles (Table 1). The specific differences in genetic profiles are detailed in the Supplementary Data (Table S1) where the distinct genetic profiles obtained from the amplification of nSSR loci for each fig tree are reported.

The number of alleles per locus (NA) ranged from 3 at locus MFC4 to 8 at locus MFC3, with an average of 5.43 alleles per locus. For the locus MFC3, the same number of alleles was observed by Ergül et al. [43] in a study on 310 fig accessions from Anatolia regions. Allele frequencies ranged from 0.034 to 0.482. The most frequent allele was allele 245 at locus LMFC31, followed by allele 173 at locus MFC2, with a frequency of 0.480, and allele 202 at locus MFC4, with a frequency of 0.476. The values for expected heterozygosity (H_E_) and observed heterozygosity (Ho) were consistently above 0.5, except for locus LMFC24, which had an Ho value of 0.400. Four loci had lower observed heterozygosity compared to expected heterozygosity, which is consistent with findings in previous studies [31,44].

The PIC (Polymorphism Information Content) value was above 0.5 for all loci analyzed, except for LMFC24. The lowest PIC observed was 0.475 for LMFC24, while the highest was 0.815 for MFC3 (Table 1). Among the primers used in this study, primers MFC2, MFC3, and LMFC30 showed the best results in terms of polymorphism, amplifying seven alleles (MFC2 and LMFC30) and eight alleles (MFC3); the performance of LMFC30 primers showed results in accordance with the study of Essid et al., where 30 fig accessions from Tunisia were analyzed [15]. These previously mentioned markers showed genetic parameters (Ho, H_E_, PIC) that were equal to or higher than those reported in previous studies where they were developed [33,42].

**Figure 1:**
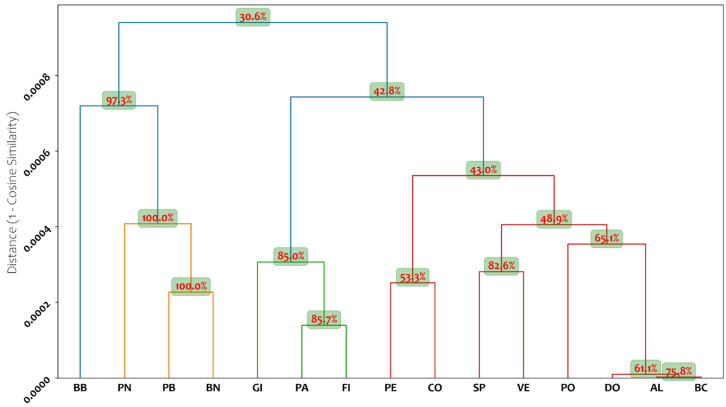
Hierarchical clustering of the analyzed cultivars based on genetic distances calculated using the cosine similarity metric and the clustering structure was derived by applying a UPGMA algorithm, which groups cultivars according to their pairwise genetic distances. The two-way (double) bootstrap support values, expressed as percentages, are displayed at each internal node, providing a measure of the stability of the corresponding clusters. AL, Albo; BB, Brogiotto Bianco; BC, Bianco di Carmignano; BN, Brogiotto Nero; CO, Corbo; DO, Dottato; FI, Fiorone; GI, Gigante di Carmignano; PA, Paradiso; PB, Pecciolo Bianco; PE, Perticone; PN, Pecciolo Nero; PO, Portogallo; SP, San Piero; VE, Verdino.

The SSR data obtained were also used to establish genetic relationships among the accessions through cluster analysis (UPGMA) using genetic distances derived from cosine similarity. Following statistical analysis, a dendrogram was produced, which enabled the evaluation of genetic similarity among the studied fig accessions (Figure 1).

In the SSR dendrogram, the first cluster contains most accessions, exhibiting different levels of genetic similarity. Within the first cluster, a larger group includes the cultivars GI, PA, FI, PE, CO, SP, VE, PO, DO, AL, and BC. This cluster can be further divided into two subgroups: the first subcluster consists of genotypes GI, PA, and FI, showing closer genetic similarity to each other than to the remaining cultivars. The second subcluster includes the remaining accessions, which can be further divided into smaller subgroups with varying genetic distances. Within this subcluster, DO, AL, and BC are the closest, suggesting high genetic similarity due to minor genetic differences between their allelic profiles. At the genetic level, a difference at two loci, LMFC30 and LMFC31, was detected between cultivar DO and cultivars AL and BC, while a difference at a single locus, LMFC30, was detected between the latter two cultivars, with a 2 bp difference in the length of one allele (Supplementary Data, Table S1). These minor genetic discrepancies may have originated from somatic mutations, which frequently occur in long-lived vegetatively propagated species [31,45]. Accessions with such characteristics might thus be considered heterogeneous clones of the same cultivar (polyclonal cultivar). However, it should be noted that these two accessions are morphologically different. The second cluster includes only four accessions: one (BB) shows a lower genetic similarity than the other three accessions (PN, PB, and BN). The genetic structure does not show any particular grouping, likely due to the narrow genetic base from which fig was domesticated, as described in previous studies [24,31].

The analysis of the hierarchical clustering of the analyzed cultivars highlights strong bootstrap support at several nodes. Highly stable clusters include BN, PB, PN (100% at each node), and BB (97.3%), followed by FI–PA (85.7%) and GI (85%). The SP and VE cluster (82.6%), AL and BC cluster (75.8%), and the DO-PO pairing (61.1% and 65.1%) show moderate-to-strong stability. However, some upper nodes in the dendrogram exhibit lower bootstrap values, particularly at higher hierarchical levels. For instance, the node joining the BB, BN, PB, and PN cluster with the rest of the dataset has a support value of only 30.6%, while other intermediate nodes, such as CO and PE (53.3%), and the node connecting it with the PO, DO, AL, and BC grouping (43.9%**)**, fall below conventional thresholds for cluster stability. This pattern suggests that while the clustering of closely related cultivars provides reliable groupings, the aggregation of more distantly related cultivars into larger clusters introduces some uncertainty and inherent variability. In particular, the reliability of clusters is greater at lower genetic distances within the dendrogram, whereas higher-level groupings exhibit reduced stability.

### 2.2. Chemical Analysis

#### 2.2.1. Identification of Phenolic Compounds

Fifteen polyphenolic compounds, belonging to major polyphenolic groups (hydroxycinnamic acids and flavonoids), and three furanocoumarins have been identified in the leaves of fig cultivars (Table 2).

**Figure 2:**
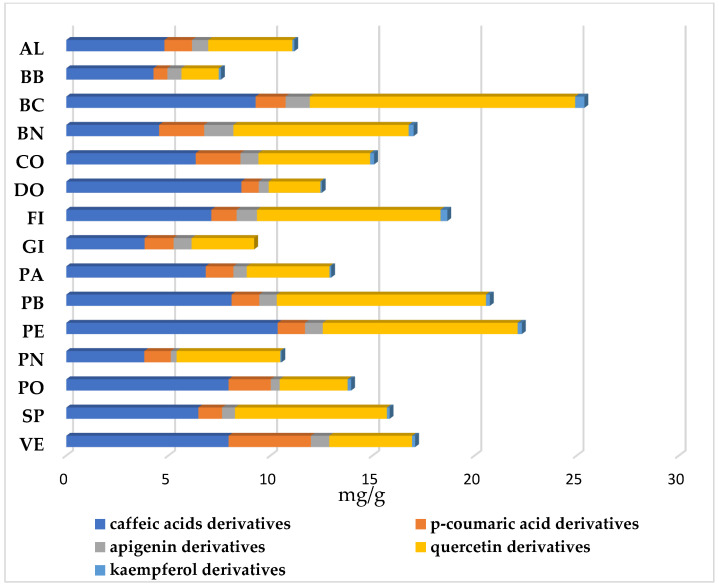
Relative distribution of phenolic and flavonoid derivatives in leaves from fig cultivars. AL, Albo; BB, Brogiotto Bianco; BC, Bianco di Carmignano; BN, Brogiotto Nero; CO, Corbo; DO, Dottato; FI, Fiorone; GI, Gigante di Carmignano; PA, Paradiso; PB, Pecciolo Bianco; PE, Perticone; PN, Pecciolo Nero; PO, Portogallo; SP, San Piero; VE, Verdino.

Generally, the same phenolic profiles were determined in each cultivar, but differences in relative levels were determined. According to previous studies, chlorogenic acid and rutin have been detected as major phenolic components in fig leaves [46,47,48,49]. Figure 2 and Table S2 show the mean values of each group of compounds found in the 15 fig cultivars.

Hydroxycinnamic acids were identified as 5-O-caffeoylquinic acid (chlorogenic acid), caffeoylmalic acid, p-coumaroyl quinic acid, and p-coumaroyl malic acid. Two other hydroxycinnamic derivatives were identified as coumaroyl and caffeoyl derivatives (Table 2); flavonoids were identified as isoschaftoside and schaftoside (apigenin derivatives), rutin, quercitin (quercetin 3-O-glucoside and quercetin 3-O-malonyl-glucoside), and kaempferol-3-glucoside (Table 2). Three other flavonoids were identified: two quercetin derivatives and a kaempferol derivative. Furanocumarins were identified as psoralen bergapten and psolaric acid (Table 2). The concentrations of caffeic acid derivatives and p-coumaroyl derivatives ranged from 2.00% to 4.51% for PA and BB, respectively, and from 0.92% to 4.17% for PE and GC, respectively, of total cultivar compounds. DO showed the highest percentage of caffeoylmalic acid, 55.85% (Table 2). DO exhibited the highest percentage of caffeic acid derivatives, 63.99%, while Brogiotto Nero had the lowest, 24.43% (Figure 2, Table S2). Globally, hydroxycinnamic acid derivatives represented 52.62% as an average of the total of phenolic compounds, varying from 36.32% of BN to almost 71.78% of VE (Figure 2, Table S2). The presence of p-coumaric acid and caffeic acid derivatives was in accordance with Ammar et al. [46] and Shiraishi et al. [47], who detected these compounds in fig leaves. As previously reported in the bibliography, the flavonoids recorded in the research mainly belonged to flavonols, such as quercetin and kaempferol derivatives, and flavones, such as apigenin (Figure 2). The cultivars presenting the highest content of flavonoid derivatives were BN with 55.00%, while VE contained only 21.89% of the total flavonoids (Table S2). Among the flavonoid compounds, rutin was the most represented, with values reaching about 40.78% in BC, while the amounts of other flavonoid compounds were low (Table 2). Our results showed that quercetin derivatives had the highest content compared to apigenin derivatives and kaempferol derivatives (Figure 2, Table S2). The average concentrations of quercetin derivatives (6.08 mg g^−1^) were 7.7 times higher than apigenin derivatives (0.79 mg g^−1^) and 35.7 times higher than kaempferol derivatives (0.18 mg g^−1^) (Table S2). BC and PB exhibited the highest percentages of quercetin derivative contents, equal to 48.64% and 47.38%, respectively, while VE and DO exhibited the lowest, with 15.89% and 18.87%, respectively. The percentages of kaempferol derivatives ranged from 1.70% in BC to 0.47% in PN, while kaempferol derivatives were absent in GI cv (Figure 2, Table S2). The total flavonoid derivatives represented 40.71% as an average of the total of phenolic compounds varying from 55.16% of BN to 21.89% of VE. The phenolic profiles of the leaves of fig cultivars in this study are in general agreement with those reported by other authors; however, there are some important differences in the absence or presence of some compounds, confirming what was reported from other authors [48,49,50]. Oliveira et al. [48] found concentrations of 5-O-caffeoylquinic acid between 1.16 and 0.47 mg g^−1^. Such values are in accordance with our results, with values ranging from 2.06 to 0.44 mg g^−1^. On the contrary, Takahashi et al. [51] reported values of rutin between 11.4 and 7.2 mg g^−1^, while our concentration for rutin ranged from 8.02 to 1.40 mg g^−1^ (Table 2). These differences in phenolic concentrations could be due to a multiplicity of factors: physiological factors and genetic factors, but even geographical location and horticultural practices [52]. The major fig leaf furanocoumarins include psoralen, bergapten, and psolaric acid isobar. The levels of furanocoumarins found in different cultivars have been reported by several authors [50,53,54,55]. Our results confirm the presence of these compounds in fig leaf. Moreover, psoralen presented the highest relative concentrations (4.87%) compared to bergapten and psolaric acid isobar (2.49% and 1.66%, respectively). Among the 15 fig cultivars, psolaren contents ranged from 8.65% in PO to 2.23% in SP (Table 2); however, their presence was not detected in GI (Table S2, Figure 2). Although furanocoumarins may cause undesirable effects in humans [51], recent evidence has suggested that these compounds possess additional biological activities, such as antioxidant and inflammatory activities [56]. Interestingly, cultivars with low or no content of psolarenic derivatives were identified in our study. These cultivars could be ideal sources of useful compounds for consumption or commercial applications.

#### 2.2.2. Total Polyphenol and Flavonoid Contents

**Figure 3:**
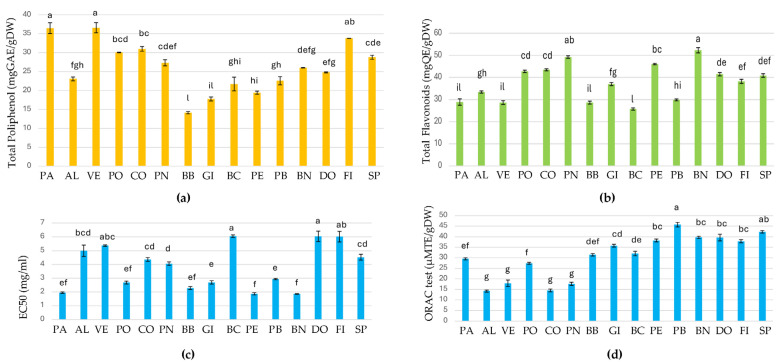
Polyphenol content (**a**), flavonoid content (**b**), EC50 (**c**), and ORAC test (**d**) in leaves of fig cultivars. Values are the means (n = 3) ± SD. Data were analyzed by ANOVA, and different letters represent significant differences (*p* < 0.05) according to post hoc comparison (Tukey’s HSD). PA, Paradiso; AL, Albo; VE, Verdino; PO, Portogallo; CO, Corbo; PN, Pecciolo Nero; BB, Brogiotto Bianco; GI, Gigante di Carmignano; BC, Bianco di Carmignano; PE, Perticone; PB, Pecciolo Bianco; BN, Brogiotto Nero; DO, Dottato; FI, Fiorone; SP, San Piero.

Total polyphenol content (TPC) by Folin–Ciocalteau assay and the total flavonoid content of fig leaves are presented in Figure 3a,b.

The TPC showed an overall mean of 25.33 mg g^−1^ DW, varying between 36.56 and 14.13 mg g^−1^ DW. No statistically significant difference was shown between VE and PA, which showed the highest values, while BB and GI had the lowest TPC (Figure 3a). Leaves from the 15 cultivars of *F. carica* could be classified according to their polyphenol content: the first group included cvs PA, VE, CO, PO, and FI, with contents greater than 30 mg g^−1^ DW. The second group, composed of PN, BN, DO, and SP, had a TPC between 24 and 30 mg g^−1^ DW, while AL, BB, GI, BC, and PB had the lowest value (between 14 and 24 mg g^−1^ DW, Figure 3a).

The total flavonoid content (TFC) ranged between 52.27 mgQEg^−1^ DW and 25.73 mgQEg^−1^ DW in fig leaf extracts, with an average value of 37.77 mgQEg^−1^ DW (Figure 3b). In terms of TFC, BN presented the highest value (52.27 mgQEg^−1^ DW), followed by the group of PN, PE, CO, PO, DO, and SP, which showed values between 40 and 50 mgQEg^−1^ DW. The other cultivars had flavonoid contents below 40 mgQEg^−1^ DW (Figure 3b). The results showed that cultivars significantly affected the TPC and TFC contents of fig leaves. The total polyphenol and flavonoid contents analyzed in this study agree with the previous work performed on fig leaves of different cultivars conducted in different areas [47,49,57].

#### 2.2.3. Antioxidant Capacity (EC50) and ORAC Test

The results of the radical scavenging capacity (DPPH) and oxygen radical absorbance capacity (ORAC) of fig leaf extracts are shown in Figure 3c,d. EC50 values ranged from 1.86 mg/mL to 6.05 mg/mL with a mean of 3.85 mg/mL. The EC50 values of the leaf extracts of fig cultivars increased in the following order: BN, PE, PA, BB, PO, GI, PB, PN, CO, SP, AL, VE, DO, and BC (Figure 3c). The total antiradicalic activity measured by the ORAC method ranged from 45.69 uMTE/gDW to 14.26 uMTE/g DW, and PB was the cultivar with the highest activity, while AL exhibited the lowest activity (Figure 3d). By comparing AA values from EC50 and ORAC (Figure 3c,d), it is evident that the methods produced different results for the cultivars analyzed. For example, PE, BN, and PA, which exhibited the highest EC50 activity (the lowest quantity of extract giving EC50 of DPPH evidences the highest efficiency), did not exhibit the highest ORAC activity, which was exhibited by SP and BB. For EC50, it is possible to assume that the highest activity of PE, BN, and PA could be due to the highest content of hydroxycinnamic derivatives and rutin. For the ORAC test, the high activity of PB and SP can be explained by a high content of hydroxycinnamic derivatives and quercetin derivatives, in particular caffeoylmalic acid and rutin, as shown in Table S2. However, these differences among AA detected by the two methods could be attributed either to differences in the specific antioxidant composition, or even to the characteristics of the analytical methods used. ORAC is an HAT-based reaction giving information on radical chain-breaking capacity, while EC50 is based on the reducing capacity of the sample [58]. For example, many ORAC-active antioxidants, such as chlorogenic acid, are not sensitive to the stable DPPH free radicals, so the DPPH assay could result in a lower antioxidant capacity [59,60]. In addition, the ORAC assay measures fluorescence to determine both the inhibition degree and time, while EC50 measures the inhibition effect at a certain time, which could be non-representative [61,62]. Since all the research cultivars were bred under the same soil and climatic conditions, the observed differences in the distribution of secondary metabolites are mainly attributable to the genotype [63]. Our study confirms the previous work evaluating fig leaves as an excellent source of bioactive compounds which can be used in the food and pharmaceutical industries [63].

For ORAC, high correlation indices (from 0.5665 to 0.9954) were found for PN, PB, PO, BC, PE, BN, DO, and SP (Table 3). All these cultivars exhibited a high concentration of caffeoyl malic, a derivative of caffeic acid and a powerful antioxidant (Table 2). FI cultivar, nevertheless, exhibits a high content of hydroxycinnamic derivatives and flavonoid derivatives, and exhibited a high correlation index of 0.7519 between ORAC and flavonoid derivatives and a correlation index of 0.7537 between EC50 and flavonoid derivatives (Table 3). Our results confirm the high scavenging and antioxidant activity of hydroxycinnamic acid derivatives and flavonoid derivatives, as reported in the literature [47,57].

#### 2.2.4. Analysis of Sugar Contents

The contents of individual and total sugars in fig cultivars are presented in Table 4. Total sugar content, calculated as the sum of fructose, glucose, and sucrose, ranged from 20.60 mg g^−1^ (BC) to 49.24 (VE), with an average of 33.97 mg g^−1^. The average concentrations of glucose, sucrose, and fructose were 1.92, 10.30, and 21.57 mg g^−1^, respectively, accounting for approximately 6%, 30%, and 64% of the total soluble sugars. The results indicate that fructose and glucose were the most abundant sugars in all fig cultivars, confirming the results of Shiraishi et al. [47]. In contrast, Vemmos et al. [63] identified sucrose as the dominant sugar in the Fracasana, Kalamon, and Mission cultivars.

The PN and VE leaf samples had the highest fructose content (mg g^−1^ DW), with values of 30.27 and 30.21, respectively, while the highest glucose content was found in VE and DO, with values of 16.86 and 15.87, respectively. Other fig leaf samples with a significant content (mg g^−1^ leaf) of fructose and glucose were Paradiso (PA; 28.92 and 15.51), Corbo (CO; 27.81 and 13.92), and Brogiotto Bianco (BB; 27.77 and 11.90), respectively.

Sucrose was present in low amounts; in fact, the average quantity determined for all cultivars was 1.89 mg g^−1^ DW. The sucrose content ranged from 1.11 mg g^−1^ DW (SP) to 3.16 mg g^−1^ DW (AL). Sucrose levels were lower in all analyzed fig cultivars compared to those reported by Teruel-Andreu et al. [55] and Shiraishi et al. [47] for the Spanish cultivar. These differences were not surprising, as a previous study on apple and fig cultivars showed significant variability in sugar concentrations due to different climate conditions and geographic regions [55,64]. It is known that sugar content is influenced by genotype but also by environmental factors and agricultural techniques. Our results showed significant variation in both total and individual sugar content. However, it is important to emphasize that the fifteen fig cultivars analyzed in this study were grown under the same soil and climatic conditions and underwent similar horticultural practices. Therefore, the observed variations in carbohydrate levels could result from a prevalent role of genetic variability in determining significant differences in sugar synthesis. Similar conclusions have been hypothesized in other species [65].

### 2.3. PCA

**Figure 4:**
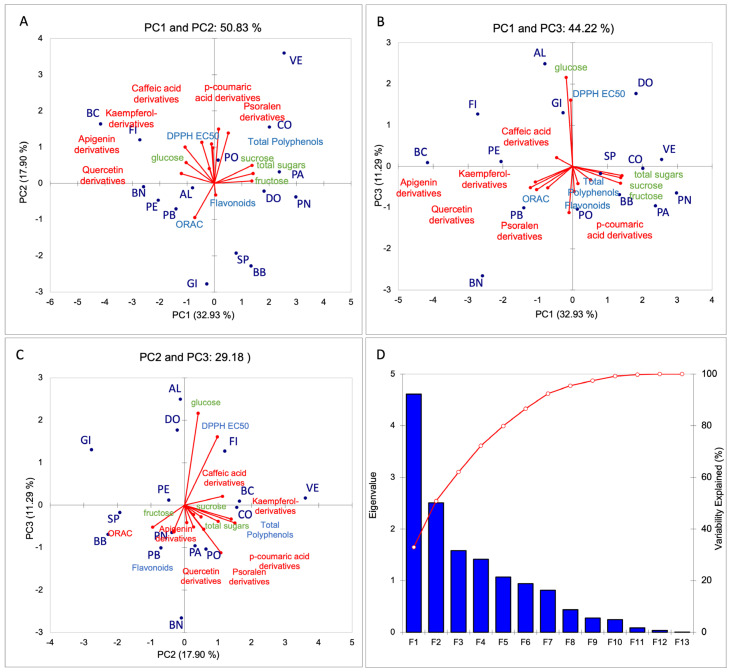
(**A**–**C**) Loading plots of the first, second, and third principal components, showing the position of fig cultivars and the different parameters studied. (**D**) Screen plot obtained from the PCA (F1–F13) denotes the principal components for the parameters studied. PA, Paradiso; AL, Albo; VE, Verdino; PO, Portogallo; CO, Corbo; PN, Pecciolo Nero; BB, Brogiotto Bianco; GI, Gigante di Carmignano; BC, Bianco di Carmignano; PE, Perticone; PB, Pecciolo Bianco; BN, Brogiotto Nero; DO, Dottato; FI, Fiorone; SP, San Piero.

The PCA score plots, along with a plot of the percentage of variance explained by 13 principal components (PCs) and their associated eigenvalues, are reported in Figure 4 and Table S3. The total variance explained by the first three principal components in the model was 61.11%. The first component (PC1) accounted for 32.93% of the total variance, the second component (PC2) for 17.90%, and the third component (PC3) for 11.23%. Only variance values greater than 0.5 were considered significant for each parameter. Sugars, including sucrose, fructose, and total sugars, showed the highest positive loading on PC1, whereas quercetin derivatives, kaempferol derivatives, and apigenin derivatives exhibited the most significant negative loading on this component. Polyphenol derivatives, such as p-coumaric acid derivatives, caffeic acid derivatives, and psoralen derivatives, showed a significant loading on PC2, with values greater than 0.500, as well as the total polyphenol parameter. PC3 demonstrated a high positive loading for glucose and antioxidant activity (DPPH EC50).

The PCA score plot showed a good distribution of samples according to their main characteristics. PC1 and PC2 plots reveal a clear separation primarily driven by sugars and certain polyphenolic derivatives. Specifically, the cultivars PA, VE, PN, DO, CO, and BB are located in the positive region of PC1 and were characterized by a higher sugar content compared to the other cultivars, such as BC, FI, BN, and PE, which are positioned in the negative region of PC1. These latter cultivars were characterized more by polyphenolic derivatives, including quercetin (quercetin derivatives at Rt 16.5 and Rt 17.9, rutin, isoquercetin), kaempferol (kaempferol 3-O-glucoside, kaempferol derivative Rt 21.2), and apigenin derivatives. Furthermore, BC and FI, positioned in the positive region of PC2, were also characterize by a higher value of p-coumaric acid, caffeic acid and psoralen derivatives, and total polyphenol content, such as VE and CO samples. In contrast, GI, BB, and AL samples were in the negative region of PC2. The cultivars AL, GI, and DO displayed distinct traits in the other two plots, linked to glucose content and antioxidant capacity (DPPH EC50), as they were positioned in the positive region of PC3. These results suggest that the chemical composition, particularly sugar and polyphenolic contents, is strongly genotype dependent, allowing clear differentiation between cultivars. Despite all samples being grown in the same field under identical conditions, significant variation was observed in the (poly)phenol content and sugar profiles of fig leaves. Similarly, Calani et al. [66] reported notable variability in the phenolic profiles of different fig peel cultivars.

## 3. Materials and Methods

### 3.1. Plant Material

The plant materials consisted of leaf samples from 15 *Ficus carica* cultivars collected from the private farm “Petracchi” located in Carmignano (Prato, Tuscany, Italy) (43°49′ N, 11°01′ E, 43°48′49′′ N, 11°1′6′′ E, 189 m. a, s. l.). Trees were cultivated under the same agro-environmental conditions and according to the standard procedures of organic cultivation. The cultivars included Albo (AL), Bianco di Carmignano (BC), Brogiotto Bianco (BB), Brogiotto Nero (BN), Corbo (CO), Dottato (DO), Fiorone (FI), Gigante di Carmignano (GI), Paradiso (PA), Pecciolo Bianco (PB), Pecciolo Nero (PN), Perticone (PE), Portogallo (PO), San Piero (SP), and Verdino (VE). Three one-year sprouts were selected from each plant. Sprouts were located on the outer part of the canopy, the sunny part. Two healthy, mature, and sunny leaves were collected from the medium part of each sprout. Samples were placed in liquid nitrogen and taken to the laboratory. The six leaves were divided into three samples. These cultivars were described in detail in a previous work [41].

### 3.2. Genetic Analysis

Total cellular DNA was extracted from fig leaves following the CTAB (Cetyl Trimethylammonium Bromide) as reported in Rodolfi et al. [31]. Seven couples of nSSR primers belonging to the MFC [42] and LMFC [33] series were used for polymorphism detection on the samples. The SSR amplification reaction was performed as reported in Rodolfi et al. [31]. The PCR amplification reaction was optimized in thermal cycler MJ PCT 100 Research (Watertown, MA, USA), programming a first passage at 94 °C for 1 min, followed by 35 cycles of 30 s at 94 °C, 30 s at the specific annealing temperature (55 °C) for each couple of primers, and 1 min at 72 °C, for denaturation, annealing, and primer extension, respectively; at the end of the cycles, they were allowed 8 min of incubation at 72 °C. The amplification products were separated with a CEQ 8000 Genetic Analysis System (Beckman Coulter, Inc., Brea, CA, USA) sequencer on acrylamide gel CEQ Separation Gel LPA-1 (Beckman Coulter, Inc., Brea, CA, USA). A marker CEQ DNA Size Standard kit 400 (Beckman Coulter, Inc., Brea, CA, USA) was used to estimate the approximate molecular weight of the amplified products. Two reference samples were used in all runs.

### 3.3. Chemical Analysis

#### 3.3.1. Phenolics Extraction

Phenolics were extracted according to Torti et al. [67]; 200 mg of lyophilized leaves were sonicated for 30 min with 5 mL of methanol 80%, centrifuged at 10,000 rpm for 10 min. Extraction was repeated thrice, and supernatants were collected. The final volume was adjusted to 10 mL. Chlorophyll was removed with an equal volume of petroleum and centrifugation at maximum speed for 5 min. Before subsequent analyses, samples were centrifuged again to remove suspended materials. Analyses were performed in triplicate. All chemicals and reagents were purchased by Merck (Darmstadt, Germany).

#### 3.3.2. HPLC-DAD-TOF-MS of Phenolics

Analyses, in triplicate, were performed as described in Petruccelli et al. [68]. The identification of phenolic compounds was performed at 280–350 nm, comparing their UV and mass spectra with the available literature and RT relative to available standards. The quantification of compounds was performed by HPLC using 5-point regression curves (R^2^ > 0.998) with authentic standards.

#### 3.3.3. Total Phenolic and Flavonoid Contents

The total phenolic content was determined with Folin–Ciocalteau reagent according to Torti et al. [67] The absorbance of the colored reaction product was read at 730 nm using a Varian UV–Visible Spectrophotometer Cary 50 Scan (Varian, MA, USA). Phenolics were expressed as mg of gallic acid equivalent per gram (mg GAE g^−1^) of dried powdered leaves, based on a standard curve. Each analysis was performed in triplicate. The flavonoid content was determined by the Aluminum Chloride test. In total, 5% Sodium Nitrite and 10% Aluminum Chloride were added to 1 mL of fig leaf extract diluted in 4 mL of ultrapure Milli Q-water (Merck, Darmastadt, Germany). The reaction was stopped with 1 mL of 1 M Sodium Hydroxide. The final volume was brought to 10 mL with Ultrapure Milli Q-water. The absorbance of the colored reaction product was read at 510 nm using a Varian UV–Visible Spectrophotometer Cary 50 Scan. Flavonoids were expressed as mg of quercetin equivalent per gram (mg QEg^−1^). Each analysis was performed in triplicate.

#### 3.3.4. Antioxidant Activity (DPPH Test)

Antioxidant activity was determined by the DPPH radical test (EC50 test) and the oxygen radical absorbance capacity (ORAC) assay. The EC50 test was performed with the DPPH radical-scavenging test according to Brand-Williams et al. [69]. The color variation in the extract (As) was measured at 517 nm after keeping the extract in the dark for 20 min. The radical scavenging activity was calculated by the percentage of scavenged DPPH, according to the following formula:

(1) $$\%\;Reduction=100\times \left(\frac{Ab-As}{Ab}\right)$$

where *Ab* is the absorbance of a blank sample, and *As* is the absorbance of leaf extracts. Ascorbic acid is the positive control. The EC50 (Effective Concentration) is the quantity of extract necessary to scavenge 50% of DPPH. Each analysis was performed in triplicate. The ORAC assay was adapted from that described by Cao and Prior [70], and the instrument was a fluorescence spectrophotometer (Varian Cary Eclipse) (Palo Alto, CA, USA). The methodology was described in Petruccelli et al. [68]. The ORAC values were expressed as uMTEg-1 of dried leaf. Each analysis was performed in triplicate.

#### 3.3.5. Analysis of Sugar Content

The freeze-dried leaf samples (30 mg) were extracted for 1 h in 3 mL of distilled water (pH 7) and centrifuged for 5 min at 10,000× *g* at 4 °C. Carbohydrate concentrations were analyzed by high-performance liquid chromatography (HPLC) according to Beghè et al. [71]. The identification and quantification of individual carbohydrates were carried out by comparing the retention times with those of authentic carbohydrate standards (Sigma-Aldrich Italia, Milan, Italy). The analyses of soluble carbohydrates were performed in triplicate, and the results were expressed as mg/g DW of fig leaf.

### 3.4. Statistical Analysis

#### 3.4.1. Molecular Analysis

For SSR analysis, fragments were sized using a conservative binning approach [72] through the statistical R 4.3.3 software (R Development Core Team 2005), which takes into account the type of replicate and compensates for the limits of fragment resolution. Genotypes showing a single allele in a given locus were indicated as homozygotes. All analyses were developed after removing duplicates, previously identified by using pairwise comparisons among all genotypes, based on their multilocus nSSR profile, using an Excel spreadsheet (Microsoft Corporation, New York, NY, USA). The information content of the SSR markers was evaluated according to the number of alleles per locus, allele frequency, observed (H_O_) and expected (H_E_) heterozygosity, the frequency of null alleles (r) [73], and polymorphic information content (PIC) [74]. Such values were obtained by using the Cervus 3.0 software [75,76]. The genetic relationships among the studied cultivars were evaluated by hierarchical clustering supported by a comprehensive bootstrap resampling analysis, which included a two-way (double) bootstrap procedure. The genetic similarities between the pairs of cultivars were initially calculated using the cosine similarity index, derived from allele size measurements across multiple genetic loci. These similarity scores were subsequently converted into genetic distances by subtracting them from unity, resulting in a distance matrix appropriate for hierarchical clustering. To prevent potential numerical inaccuracies, diagonal values in this matrix were explicitly set to zero. Hierarchical clustering was then carried out using the Unweighted Pair Group Method with arithmetic mean (UPGMA), selected for its ability to effectively represent hierarchical relationships among genetic samples. To rigorously assess the stability and reliability of the obtained dendrogram, a double bootstrap approach was implemented, involving simultaneous resampling along both the rows (cultivars) and columns (genetic loci). Specifically, this two-way bootstrap procedure consisted of 300 iterations, each generating a new dataset by independently resampling both the cultivars and the genetic loci with replacements. In each iteration, clustering was performed anew, and the co-occurrences of the pairs of cultivars within clusters were recorded, cumulatively forming a co-occurrence matrix. This matrix quantified how frequently each pair of cultivars appeared together within the same cluster across all iterations. Consequently, the frequency of these co-occurrences provided bootstrap percentages, indicative of the robustness of each cluster. The stability percentages obtained from the double bootstrap were subsequently annotated directly onto the dendrogram, facilitating the immediate visual interpretation of cluster reliability. This integrative double bootstrap methodology thereby provides an enhanced and statistically comprehensive assessment of genetic relationships among studied cultivars. To produce dendrograms and the implementation of bootstrap analysis, we developed a dedicated code in the Anaconda environment [77] and Python ecosystem using dedicated scientific python libraries, such as Pandas [78], Scipy (for hierarchical clustering analysis) [79], Statsmodels [80], and Pingouin [81]. Plots were derived using Seaborn [82] and MatplotLib [83] Python modules.

#### 3.4.2. Chemical Analysis

Chemical analysis data were tested for differences between cultivars using the one-way analysis of variance (ANOVA). Differences were tested with Tukey’s high significance difference (HSD) test at the 0.05 significance level (Statgraphics Plus, version 5.1 for Windows). Data are presented as mean +/− standard deviation (SD). The dataset underwent a principal component analysis (PCA) to better assess the potential association among the samples and measured parameters. The analysis was performed using XLSTAT 2024 software [84].

## 4. Conclusions

In the present study, 15 cultivars of *Ficus carica*, grown at the same site, were evaluated for varietal identification using molecular and biochemical markers. The results revealed significant variability among cultivars based on these markers. SSR analysis produced distinct genotypic profiles for each cultivar, with cluster analysis (UPGMA) grouping the 15 cultivars into two main clusters. In particular, the Dottato (DO), Albo (AL), and Bianco di Carmignano (BC) cultivars showed high genetic similarity, likely due to somatic mutations in long-lived trees propagated vegetatively or the presence of polyclonal cultivars, a phenomenon previously observed in other species [85].

The phytochemical profiling of the leaves, conducted by HPLC-DAD-TOF-MS, revealed 17 phenolic compounds, among which the most representative were caffeic acid derivatives. The psoralen compound was absent only in Gigante di Carmignano cv. Fig cultivars differ in the total concentration of chemical attributes and antioxidant activities, which appear to be cultivar specific. Among the 15 cultivars analyzed, Paradiso (PA) and Verdino (VE) can be considered very interesting for their total polyphenol content, Pecciolo Nero (PN) and Perticone (PE) for their total flavonoid content, and Gigante di Carmignano (GC) for the absence of psolaren derivatives.

The complete genetic and chemical characterization of the 15 fig cultivars studied offers valuable insights for both conservation strategies and agricultural applications. This multidisciplinary approach allows for a more accurate and realistic analysis, providing valuable information for the enhancement and conservation of local genetic resources.

## Figures and Tables

**Table 1:** Allele size (base pairs), number of alleles (NA) per locus, expected heterozygosity (H_E_), observed heterozygosity (Ho), estimated frequency of null alleles (r), and polymorphic information content (PIC) in the total sample.

	Locus	Locus	Locus	Locus	Locus	Locus	Locus
	MFC2	MFC3	MFC4	LMFC12	LMC24	LMFC30	LMFC31
a	159	124	202	353	270	236	229
b	161	126	222	372	276	246	231
c	167	128	226	375	278	252	243
d	169	130		381	280	258	245
e	171	132		401		260	
f	173	136				262	
g	179	138				266	
h		144					
NA	7	8	3	5	4	7	4
H_E_	0.729	0.864	0.687	0.639	0.563	0.855	0.634
H_O_	0.800	0.733	0.800	0.600	0.400	0.733	0.667
r	−0.070	0.053	−0.104	0.018	0.188	0.063	−0.042
PIC	0.661	0.815	0.590	0.542	0.475	0.803	0.537

**Table 2:** Compounds’ concentrations in leaves of 15 fig cultivars (mg/g DW). Hydroxycinnamic acids’ individual compounds, flavonoid derivatives’ individual compounds, and psoralen derivatives’ individual compounds.

**CV**	**5-OCaffeoylquinic Acid**	**Caffeoylmalic ACID**	**Caffeic Acid ** **Derivatives**	**p-Coumaroyl Derivative**	**p-Coumaroylquinic Acid**	**p-Coumaroyl ** **Malic Acid**	**Psolaren**	**Bergapten ** **(5-methoxypsolaren)**	**Psolaric Acid Isobar**
	**mg/g DW**	**mg/g DW**	**mg/g DW**	**mg/g DW**	**mg/g DW**	**mg/g DW**	**mg/g DW**	**mg/g DW**	**mg/g DW**
PA	0.550 ± 0.04	5.963 ± 0.39	0.284 ± 0.02	0.426 ± 0.03	0.555 ± 0.04	0.367 ± 0.03	0.786 ± 0.06	0.283 ± 0.02	0.272 ± 0.02
AL	0.566 ± 0.03	3.847 ± 0.25	0.391 ± 0.03	0.324 ± 0.02	1.063 ± 0.08	0.672 ± 0.05	0.667 ± 0.05	0.383 ± 0.04	0.318 ± 0.02
VE	0.563 ± 0.04	6.712 ± 0.48	0.675 ± 0.04	nd	2.646 ± 0.17	1.385 ± 0.09	0.729 ± 0.05	0.445 ± 0.03	0.319 ± 0.03
PO	0.745 ± 0.05	9.221 ± 0.66	0.543 ± 0.05	nd	0.462 ± 0.03	0.364 ± 0.03	1.488 ± 0.11	0.614 ± 0.08	0.381 ± 0.03
CO	0.479 ± 0.03	5.314 ± 0.36	0.552 ± 0.04	nd	1.222 ± 0.08	0.972 ± 0.07	0.760 ± 0.07	0.451 ± 0.03	0.357 ± 0.04
PN	0.561 ± 0.04	2.784 ± 0.22	0.465 ± 0.03	0.397 ± 0.03	0.513 ± 0.04	0.394 ± 0.04	0.888 ± 0.06	0.420 ± 0.03	0.407 ± 0.03
BB	0.764 ± 0.05	3.124 ± 0.23	0.385 ± 0.04	nd	0.217 ± 0.02	0.466 ± 0.05	0.673 ± 0.05	0.581 ± 0.05	0.278 ± 0.02
GI	0.444 ± 0.03	3.391 ± 0.24	nd	0.384 ± 0.03	0.467 ± 0.04	0.569 ± 0.04	nd	nd	nd
BC	2.059 ± 0.14	6.674 ± 0.45	0.533 ± 0.04	0.764 ± 0.06	0.363 ± 0.03	0.344 ± 0.03	0.948 ± 0.08	0.354 ± 0.03	0.382 ± 0.04
PE	1.875 ± 0.13	7.837 ± 0.57	0.620 ± 0.04	0.213 ± 0.02	0.282 ± 0.02	0.842 ± 0.06	0.861 ± 0.06	0.283 ± 0.02	0.182 ± 0.01
PB	0.759 ±0.05	6.843 ± 0.47	0.485 ± 0.04	0.314 ± 0.02	0.372 ± 0.05	0.671 ± 0.05	0.683 ± 0.05	0.262 ± 0.02	0.177 ± 0.02
BN	0.821 ± 0.06	3.257 ± 0.06	0.454 ± 0.03	nd	1.426 ± 0.09	0.784 ± 0.05	1.158 ± 0.09	0.593 ± 0.04	0.416 ± 0.05
DO	0.558 ± 0.04	7.483 ± 0.52	0.530 ± 0.04	nd	0.461 ± 0.03	0.386 ± 0.03	0.883 ± 0.06	0.529 ± 0.04	nd
FI	0.884 ± 0.06	5.759 ± 0.25	0.462 ± 0.30	0.474 ± 0.03	0.384 ± 0.03	0.380 ± 0.02	0.696 ± 0.05	0.342 ± 0.02	0.355 ± 0.04
SP	0.986 ± 0.07	4.888 ± 0.35	0.590 ± 0.04	0.450 ± 0.04	0.364 ± 0.04	0.347 ± 0.02	0.359 ± 0.03	nd	nd
**CV**	**Isoschaftoside**	**Schaftoside**	**Quercetin Derivative ** **(Rt 16.5)**	**Quercetin Derivative (Rt 17.9)**	**Rutin (quercetin-3-O-rutinoside)**	**Isoquercitin (quercetin ** **3-O-glucoside)**	**Quercetin ** **3-O-malonyl-glucoside**	**Kaempferol ** **3-O-glucoside**	**Kaempferol ** **Derivative (Rt 21.2)**
PA	0.486 ± 0.03	0.161 ± 0.01	0.135 ± 0.01	0.095 ± 0.01	3.568 ± 0.27	0.074 ± 0.01	0.184 ± 0.02	0.080 ± 0.02	nd
AL	0.555 ± 0.03	0.237 ± 0.02	0.379 ± 0.03	0.184 ± 0.02	3.333 ± 0.25	0.078 ± 0.02	0.146 ± 0.01	0.095 ± 0.01	nd
VE	0.665 ± 0.04	0.231 ± 0.01	nd	nd	2.364 ± 0.16	0.067 ± 0.01	0.171 ± 0.01	0.153 ± 0.02	nd
PO	0.284 ± 0.02	0.189 ± 0.01	0.071 ± 0.01	0.068 ± 0.01	2.518 ± 0.18	0.120 ± 0.03	0.549 ± 0.04	0.170 ± 0.01	nd
CO	0.654 ± 0.04	0.223 ± 0.01	nd	nd	4.887 ± 0.32	0.193 ± 0.01	0.394 ± 0.03	0.195 ± 0.03	nd
PN	0.137 ± 0.01	0.145 ± 0.01	0.164 ± 0.01	0.084 ± 0.02	3.728 ± 0.25	0.577 ± 0.04	0.526 ± 0.03	0.055 ± 0.01	nd
BB	0.455 ± 0.03	0.234 ± 0.02	0.063 ± 0.01	0.043 ± 0.01	1.397 ± 0.10	0.179 ± 0.01	0.142 ± 0.01	0.110 ± 0.02	nd
GI	0.535 ± 0.04	0.343 ± 0.03	0.457 ± 0.03	0.161 ± 0.02	2.117 ± 0.15	0.132 ± 0.01	0.194 ± 0.02	nd	nd
BC	0.654 ± 0.05	0.528 ± 0.04	0.236 ± 0.02	0.226 ± 0.02	8.238 ± 0.58	1.625 ± 0.12	2.668 ± 0.18	0.394 ± 0.03	0.053 ± 0.01
PE	0.513 ± 0.04	0.355 ± 0.02	0.210 ± 0.01	0.186 ± 0.03	7.247 ± 0.50	0.652 ± 0.04	1.243 ± 0.13	0.208 ± 0.02	nd
PB	0.489 ± 0.03	0.368 ± 0.03	0.255 ± 0.02	0.127 ± 0.01	8.020 ± 0.55	1.183 ± 0.08	0.658 ± 0.05	0.193 ± 0.01	nd
BN	0.996 ± 0.07	0.434 ± 0.03	0.144 ± 0.01	0.054 ± 0.01	7.583 ± 0.53	0.415 ± 0.03	0.385 ± 0.03	0.242 ± 0.02	nd
DO	0.293 ± 0.02	0.195 ± 0.01	0.054 ± 0.01	0.082 ± 0.02	1.655 ± 0.12	0.153 ± 0.01	0.585 ± 0.04	0.069 ± 0.02	nd
FI	0.556 ± 0.04	0.442 ± 0.03	0.306 ± 0.02	0.194 ± 0.03	5.784 ± 0.39	0.962 ± 0.07	1.741 ± 0.12	0.313 ± 0.02	0.021 ± 0.02
SP	0.399 ± 0.03	0.232 ± 0.02	0.098 ± 0.01	0.075 ± 0.01	5.661 ±0.41	0.631 ± 0.05	0.973 ± 0.07	0.134 ± 0.01	nd

Values (mean ± standard error; n = 3). nd: not determined. PA, Paradiso; AL, Albo; VE, Verdino; PO, Portogallo; CO, Corbo; PN, Pecciolo Nero; BB, Brogiotto Bianco; GI, Gigante di Carmignano; BC, Bianco di Carmignano; PE, Perticone; PB, Pecciolo Bianco; BN, Brogiotto Nero; DO, Dottato; FI, Fiorone; SP, San Piero.

**Table 3:** Correlation indices calculated between hydroxycinnamic derivative and flavonoid derivative concentrations vs. ORAC and EC50 values. AL, Albo; BB, Brogiotto Bianco; BC, Bianco di Carmignano; BN, Brogiotto Nero; CO, Corbo; DO, Dottato; FI, Fiorone; GI, Gigante di Carmignano; PA, Paradiso; PB, Pecciolo Bianco; PE, Perticone; PN, Pecciolo Nero; PO, Portogallo; SP, San Piero; VE, Verdino.

CV	Hy.Der./EC50	Hy.Der/ORAC	Fl. Der./EC50	Fl.Der./ORAC
PA	0.1293	0.1479	0.1731	0.1940
AL	0.3912	0.3918	0.0431	0.0434
VE	0.4323	0.4023	0.9556	0.9423
BC	0.9643	0.9637	0.8322	0.8334
BB	0.3007	0.2865	0.0005	0.0015
BN	0.8338	0.7709	0.3046	0.3796
CO	0.0332	0.0291	0.9912	0.9932
DO	0.8355	0.8330	0.4330	0.4296
FI	0.2927	0.2947	0.7537	0.7519
GI	0.4453	0.4453	0.9494	0.9494
PB	0.6460	0.6823	0.0845	0.1071
PN	0.9868	0.9873	0.7500	0.7519
PE	0.5819	0.5594	0.1824	0.1805
PO	0.9935	0.9954	0.8818	0.8736
SP	0.5715	0.5665	0.3001	0.3048

**Table 4:** Sugar contents (mg g^−1^ DW) in mature leaves of fig cultivars.

CV	Fructose	Glucose	Sucrose	Total Sugars
AL	14.19 ± 0.38 ^h^	6.36 ± 0.14 ^i^	3.16 ± 0.08 ^a^	23.72 ± 0.43 ^i^
BB	27.77 ± 0.22 ^c^	11.90 ± 0.16 ^e^	1.21 ± 0.03 ^de^	40.88 ± 0.39 ^e^
BC	13.82 ± 0.21 ^hi^	5.22 ± 0.12 ^j^	1.55 ± 0.07 ^c^	20.60 ± 0.16 ^k^
BN	14.23 ± 0.26 ^h^	6.49 ± 0.17 ^hi^	1.18 ± 0.05 ^de^	21.91 ± 0.25 ^j^
CO	27.81 ± 0.27 ^c^	13.92 ± 0.12 ^c^	2.05 ± 0.06 ^b^	43.74 ± 0.22 ^d^
DO	24.26 ± 0.30 _d_	12.90 ± 0.17 ^d^	2.96 ± 0.04 ^ab^	43.15 ± 0.14 ^e^
FI	13.31 ± 0.24 ^i^	6.24 ± 0.18 ^i^	2.76 ± 0.09 ^b^	22.31 ± 0.28 ^j^
GI	18.78 ± 0.25 ^f^	6.81 ± 0.16 ^h^	2.30 ± 0.05 ^c^	27.89 ± 0.40 ^h^
PA	28.92 ± 0.25 ^b^	15.51 ± 0.15 ^b^	1.50 ± 0.04 ^c^	45.70 ± 0.18 ^c^
PB	19.66 ± 0.35 ^e^	10.11 ± 0.1 ^f1^	1.13 ± 0.08 ^e^	30.90 ± 0.36 ^g^
PE	18.69 ± 0.30 ^f^	6.92 ± 0.14 ^h^	2.77 ± 0.08 ^b^	28.38 ± 0.34 ^h^
PN	30.27 ± 0.26 ^a^	15.56 ± 0.15 ^b^	1.43 ± 0.06 ^cd^	47.26 ± 0.44 ^b^
PO	16.69 ± 0.28 ^g^	9.53 ± 0.17 ^g^	1.51 ± 0.05 ^c^	27.73 ± 0.17 ^h^
SP	24.91 ± 0.30 ^d^	10.24 ± 0.13 ^f^	1.11 ± 0.03 ^e^	36.27 ± 0.36 ^f^
VE	30.21 ± 0.28 ^a^	16.86 ± 0.10 ^a^	2.12 ± 0.09 ^b^	49.24 ± 0.25 ^a^

Values (mean ± standard error; n = 3) followed by the same letter, within the same column, are not significantly different (*p* > 0.05), according to Tukey’s least significant difference test. AL, Albo; BB, Brogiotto Bianco; BC, Bianco di Carmignano; BN, Brogiotto Nero; CO, Corbo; DO, Dottato; FI, Fiorone; GI, Gigante di Carmignano; PA, Paradiso; PB, Pecciolo Bianco; PE, Perticone; PN, Pecciolo Nero; PO, Portogallo; SP, San Piero; VE, Verdino.

## Data Availability

All data will be provided upon request to the authors.

## Supplementary Materials

The following supporting information can be downloaded at: https://www.mdpi.com/article/10.3390/plants14081238/s1, Table S1: Genetic profiles of 15 fig cultivars; Table S2: Chemical compounds concentrations in leaves of 15 fig cultivars (mg/g DW); Table S3: Correlation coefficients between the main PCA components.

## References

[1] Falistocco E. (2024). The World of Figs: An Overview. Economically Important Trees: Origin, Evolution, Genetic Diversity and Ecology.

[2] Zohary D., Hopf M., Weiss E. (2012). Domestication of Plants in the Old World: The Origin and Spread of Domesticated Plants n SouthwesAsia, Europe, and the Mediterranean Basin.

[3] Goldschmidt E.E. (2013). The Evolution of Fruit Tree Productivity: A Review. Econ. Bot..

[4] Falistocco E. (2020). The Millenary History of the Fig Tree (*Ficus carica* L.). Adv. Agric. Hortic. Entomol..

[5] Mazzeo A., Magarelli A., Ferrara G. (2024). The Fig (*Ficus carica* L.): Varietal Evolution from Asia to Puglia Region, Southeastern Italy. CABI Agric. Biosci..

[6] Nosir W., Ramadan M.F. (2023). Cultivars and Agriculture Practice of Fig (*Ficus carica*). Fig (Ficus carica): Production, Processing and Properties.

[7] Hajam T.A., Saleem H. (2022). Phytochemistry, Biological Activities, Industrial and Traditional Uses of Fig (*Ficus carica*): A Review. Chem. Biol. Interact..

[8] Nawaz H., Waheed R., Nawaz M. (2020). Phytochemical Composition, Antioxidant Potential, and Medicinal Significance of Ficus. Mod. Fruit. Ind..

[9] Khadivi A., Mirheidari F. (2023). Phenotypic Variability of Fig (*Ficus carica* L.). Fig (Ficus carica): Production, Processing, and Properties.

[10] Mafrica R., De Bruno A., Piscopo A., Poiana M., Bruno M., Caruso T. (2021). Cultivar and Accessions of Fig (*Ficus carica* L.) for Breba Production Selected within the Autochthonous Germplasm of Calabria (South Italy). Acta Hortic..

[11] Nuzzo V., Gatto A., Montanaro G. (2022). Morphological Characterization of Some Local Varieties of Fig (*Ficus carica* L.) Cultivated in Southern Italy. Sustainability.

[12] Ayar A., Şahin B., Mutlu D., Özen M., Belge A., Karacaoğlan Ç. (2023). Fig (*Ficus carica* var. *domestica* L.) Genetic resources Conservation and characterization. Uluslararası Doğu Anadolu Fen Mühendislik Ve Tasarım Dergisi.

[13] Uğur R., Gündeşli M.A., Özatar H.O., Özen M., Aras S. (2023). Determination of Fruit Characteristics of Some Fig Genotypes (*Ficus carica* L.) Obtained by Selection Breeding in the Eastern Mediterranean Region. Int. J. Agric. Environ. Food Sci..

[14] Gaaliche B., Saddoud O., Mars M. (2012). Morphological and Pomological Diversity of Fig (*Ficus carica* L.) Cultivars in Northwest of Tunisia. Int. Sch. Res. Not..

[15] Essid A., Aljane F., Neily M.H., Ferchichi A., Hormaza J.I. (2021). Assessment of Genetic Diversity of Thirty Tunisian Fig (*Ficus carica* L.) Accessions Using Pomological Traits and SSR Markers. Mol. Biol. Rep..

[16] Perez-Jiménez M., López B., Dorado G., Pujadas-Salvá A., Guzmán G., Hernandez P. (2012). Analysis of Genetic Diversity of Southern Spain Fig Tree (*Ficus carica* L.) and Reference Materials as a Tool for Breeding and Conservation. Hereditas.

[17] Núñez-Gómez D., Legua P., Martínez-Nicolás J.J., Melgarejo P. (2021). Breba Fruits Characterization from Four Varieties (*Ficus carica* L.) with Important Commercial Interest in Spain. Foods.

[18] Giraldo E., López-Corrales M., Hormaza J.I. (2010). Selection of the most discriminating morphological qualitative variables for characterization of fig germplasm. J. Am. Soc. Hort. Sci..

[19] Ciarmiello L.F., Piccirillo P., Carillo P., De Luca A., Woodrow P. (2015). Determination of the Genetic Relatedness of Fig (*Ficus carica* L.) Accessions Using RAPD Fingerprint and Their Agro-Morphological Characterization. S. Afr. J. Bot..

[20] De Masi L., Castaldo D., Galano G., Minasi P., Laratta B. (2005). Genotyping of Fig (*Ficus carica* L) via RAPD Markers. J. Sci. Food Agric..

[21] Salhi Hannachi A., Chatti K., Marrakchi M., Trifi M., Mars M. (2003). Specific Genetic Markers for Tunisian Fig Germplasm: Evidence of Morphological Traits, Random Amplified Polymorphic DNA and Inter Simple Sequence Repeats Markers [RAPD; ISSR; *Ficus carica* L.]. J. Genet. Breed. Italy.

[22] Qurbanova Q., Babayeva S., Abbasov M. (2025). Analysis of the Genetic Diversity of Azerbaijani Fig Accessions (*Ficus carica* L.) Using Pomological Traits and Inter Simple Sequence Repeat (ISSR) Markers. Genet. Resour. Crop Evol..

[23] Uçer V.A., Aglar E., Mortazavi P., Qureshi S.A., Ali A., Tatar M., Altaf M.T., Bedir M., Ercişli S., Nadeem M.A. (2024). Exploring Genetic Diversity of Turkish Fig (*Ficus carica* L.) Germplasm Using Inter-Primer Binding Site (iPBS) Retrotransposon Markers. Genet. Resour. Crop Evol..

[24] Sclavounos A., Roussos P., Milla S., Kostas P., Samaras Y., Pozzi C., Molla J., Chitikineni A., Varshney R.K., Voloudakis A. (2023). Genetic Diversity of Fig (*Ficus carica* L.) Germplasm from the Mediterranean Basin as Revealed by SSR Markers. Genet. Resour. Crop Evol..

[25] Ganopoulos I., Xanthopoulou A., Molassiotis A., Karagiannis E., Moysiadis T., Katsaris P., Aravanopoulos F., Tsaftaris A., Kalivas A., Madesis P. (2015). Mediterranean Basin *Ficus carica* L.: From Genetic Diversity and Structure to Authentication of a Protected Designation of Origin Cultivar Using Microsatellite Markers. Trees.

[26] Akin M., Poljuha D., Eyduran S.P., Ercisli S., Radunic M. (2021). SSR Based Molecular Characterization of Local Fig (*Ficus carica* L.) Germplasm in Northeastern Turkey. Erwerbs-Obstbau.

[27] Belttar H., Yahia A., Nemli S., Ates D., Erdogmus S., Ertan B., Himour S., Hepaksoy S., Tanyolac M.B. (2017). Determination of the Population Structure of Fig Genotypes from Algeria and Turkey Using Inter Primer Binding Site-Retrotransposon and Simple Sequence Repeat Markers. Agric. Sci..

[28] Baraket G., Chatti K., Saddoud O., Abdelkarim A.B., Mars M., Trifi M., Hannachi A.S. (2011). Comparative Assessment of SSR and AFLP Markers for Evaluation of Genetic Diversity and Conservation of Fig, *Ficus carica* L., Genetic Resources in Tunisia. Plant Mol. Biol. Rep..

[29] Haffar S., Baraket G., Usai G., Aounallah A., Ben Mustapha S., Ben Abdelkrim A., Salhi Hannachi A. (2022). Conserved DNA-Derived Polymorphism as a Useful Molecular Marker to Explore Genetic Diversity and Relationships of Wild and Cultivated Tunisian Figs (*Ficus carica* L.). Trees.

[30] Boudchicha R., Hormaza J., Benbouza H. (2018). Diversity Analysis and Genetic Relationships among Local Algerian Fig Cultivars (*Ficus carica* L.) Using SSR Markers. S. Afr. J. Bot..

[31] Rodolfi M., Ganino T., Chiancone B., Petruccelli R. (2018). Identification and Characterization of Italian Common Figs (*Ficus carica*) Using Nuclear Microsatellite Markers. Genet. Resour. Crop Evol..

[32] Ferrara G., Mazzeo A., Pacucci C., Matarrese A.M.S., Tarantino A., Crisosto C., Incerti O., Marcotuli I., Nigro D., Blanco A. (2016). Characterization of Edible Fig Germplasm from Puglia, Southeastern Italy: Is the Distinction of Three Fig Types (Smyrna, San Pedro and Common) Still Valid?. Sci. Hortic..

[33] Giraldo E., López-Corrales M., Viruel M., Hormaza J. Development of Microsatellite Markers in Fig (*Ficus carica* L.). Proceedings of the XI Eucarpia Symposium on Fruit Breeding and Genetics.

[34] Ramawat K.G. (2019). An Introduction to Biodiversity and Chemotaxonomy. Biodivers. Chemotaxon..

[35] Peters K., Blatt-Janmaat K.L., Tkach N., van Dam N.M., Neumann S. (2023). Untargeted Metabolomics for Integrative Taxonomy: Metabolomics, DNA Marker-Based Sequencing, and Phenotype Bioimaging. Plants.

[36] Pagare S., Bhatia M., Tripathi N., Pagare S., Bansal Y. (2015). Secondary Metabolites of Plants and Their Role: Overview. Curr. Trends Biotechnol. Pharm..

[37] Misra A., Srivastava S. (2016). Chemotaxonomy: An Approach for Conservation and Exploration of Industrially Potential Medicinal Plants. J. Pharmacogn. Nat. Prod..

[38] Mannheimer C.A. (1999). An Overview of Chemotaxonomy and Its Role in Creating a Phylogenetic Classification System. Natl. Bot. Res. Inst. Minist. Agric. Water Rural Dev. Windhoek.

[39] Ankanna S., Suhrulatha D., Savithramma N. (2012). Chemotaxonomical Studies of Some Important Monocotyledons. Bot. Res. Int..

[40] Oladipo O.T., Akinpelu B.A., Folorunso A.E., Godwin A., Omotoso S.E., Dosunmu O.A., Joseph W.A. (2017). Chemotaxonomic Study of Six Nigerian Ficus Species (Moraceae). Not. Sci. Biol..

[41] 41 Giordano C., Arcidiaco L., Rodolfi M., Ganino T., Beghè D., Petruccelli R. (2025). Description of *Ficus carica* L. Italian Cultivars—I: Machine Learning Based Analysis of Leaf Morphological Traits. Plants.

[42] Khadari B., Hochu I., Santoni S., Kjellberg F. (2001). Identification and Characterization of Microsatellite Loci in the Common Fig (*Ficus carica* L.) and Representative Species of the Genus Ficus. Mol. Ecol. Notes.

[43] Ergül A., Büyük B.P., Hazrati N., Yılmaz F., Kazan K., Arslan N., Özmen C.Y., Aydın S.S., Bakır M., Tan N. (2021). Genetic Characterisation and Population Structure Analysis of Anatolian Figs (*Ficus carica* L.) by SSR Markers. Folia Hortic..

[44] Saddoud O., Chatti K., Salhi-Hannachi A., Mars M., Rhouma A., Marrakchi M., Trifi M. (2007). Genetic Diversity of Tunisian Figs (*Ficus carica* L.) as Revealed by Nuclear Microsatellites. Hereditas.

[45] Petruccelli R., Beghè D., Ganino T., Bartolini G., Ciaccheri L., Bernardi R., Durante M. (2020). Evaluation of Intra-Cultivar Variability in’*Olea europaea*’ L. Cv. Leccino Using Morphological, Biochemical and Molecular Markers. Aust. J. Crop Sci..

[46] Ammar S., Contreras M.D.M., Belguith-Hadrich O., Bouaziz M., Segura-Carretero A. (2015). New Insights into the Qualitative Phenolic Profile of *Ficus carica* L. Fruits and Leaves from Tunisia Using Ultra-High-Performance Liquid Chromatography Coupled to Quadrupole-Time-of-Flight Mass Spectrometry and Their Antioxidant Activity. RSC Adv..

[47] Shiraishi C.S.H., Zbiss Y., Roriz C.L., Dias M.I., Prieto M.A., Calhelha R.C., Alves M.J., Heleno S.A., V. d.C.M., Carocho M. (2023). Fig Leaves (*Ficus carica* L.): Source of Bioactive Ingredients for Industrial Valorization. Processes.

[48] Oliveira A.P., Valentão P., Pereira J.A., Silva B.M., Tavares F., Andrade P.B. (2009). *Ficus carica* L.: Metabolic and Biological Screening. Food Chem. Toxicol..

[49] Akhtar P., Yaakob Z., Ahmed Y., Shahinuzzaman M., del Mar Contreras M. (2019). Potential of Leaves of Eighteen Cultivars of *Ficus carica* as Antioxidants and Profiling of Phenolic Compounds as an Active Molecules: Antioxidant Active Compounds Isolation from the Leaves of *Ficus carica* (Cv. Violette Solise. Iran. J. Pharm. Sci..

[50] Takahashi T., Okiura A., Kohno M. (2017). Phenylpropanoid Composition in Fig (*Ficus carica* L.) Leaves. J. Nat. Med..

[51] Takahashi T., Okiura A., Saito K., Kohno M. (2014). Identification of Phenylpropanoids in Fig (*Ficus carica* L.) Leaves. J. Agric. Food Chem..

[52] Bruni R., Sacchetti G. (2009). Factors Affecting Polyphenol Biosynthesis in Wild and Field Grown St. John’s Wort (*Hypericum perforatum* L. Hypericaceae/Guttiferae). Molecules.

[53] Imen M., Ahmadabadi A., Tavousi S., Sedaghat A. (2019). The Curious Cases of Burn by Fig Tree Leaves. Indian J. Dermatol..

[54] Zhang Y., Wan Y., Huo B., Li B., Jin Y., Hu X. (2018). Extracts and Components of *Ficus carica* Leaves Suppress Survival, Cell Cycle, and Migration of Triple-Negative Breast Cancer MDA-MB-231 Cells. OncoTargets Ther..

[55] Teruel-Andreu C., Andreu-Coll L., López-Lluch D., Sendra E., Hernández F., Cano-Lamadrid M. (2021). *Ficus carica* Fruits, by-Products and Based Products as Potential Sources of Bioactive Compounds: A Review. Agronomy.

[56] Del Río J.A., Díaz L., García-Bernal D., Blanquer M., Ortuno A., Correal E., Moraleda J.M. (2014). Furanocoumarins: Biomolecules of Therapeutic Interest. Stud. Nat. Prod. Chem..

[57] Ben Achour Harrabi N., Taamalli W., Jiljli H., Dlima I., Yangui I., Hachicha D., Attia R., Mejri M. (2024). Nutritional Profile, Phytochemical Characterization, and Biological Activities of Tunisian Cultivar *Ficus carica* Zidi Leaves. Euro-Mediterr. J. Environ. Integr..

[58] Prior R.L., Wu X., Schaich K. (2005). Standardized Methods for the Determination of Antioxidant Capacity and Phenolics in Foods and Dietary Supplements. J. Agric. Food Chem..

[59] Huang D., Ou B., Prior R.L. (2005). The Chemistry behind Antioxidant Capacity Assays. J. Agric. Food Chem..

[60] Lohachoompol V., Mulholland M., Srzednicki G., Craske J. (2008). Determination of Anthocyanins in Various Cultivars of Highbush and Rabbiteye Blueberries. Food Chem..

[61] Brambilla A., Lo Scalzo R., Bertolo G., Torreggiani D. (2008). Steam-Blanched Highbush Blueberry (*Vaccinium corymbosum* L.) Juice: Phenolic Profile and Antioxidant Capacity in Relation to Cultivar Selection. J. Agric. Food Chem..

[62] Wang Y., Liu X., Chen S., Wang Q., Jin B., Wang L. (2024). Functions, Accumulation, and Biosynthesis of Important Secondary Metabolites in the Fig Tree (*Ficus carica*). Front. Plant Sci..

[63] Vemmos S.N., Petri E., Stournaras V. (2013). Seasonal Changes in Photosynthetic Activity and Carbohydrate Content in Leaves and Fruit of Three Fig Cultivars (*Ficus carica* L.). Sci. Hortic..

[64] Cvetković B., Bajić A., Belović M., Pezo L., Dragojlović D., Šimurina O., Djordjević M., Korntheuer K., Philipp C., Eder R. (2024). Assessing Antioxidant Properties, Phenolic Compound Profiles, Organic Acids, and Sugars in Conventional Apple Cultivars (*Malus domestica*): A Chemometric Approach. Foods.

[65] Beccaro G.L., Donno D., Lione G.G., De Biaggi M., Gamba G., Rapalino S., Riondato I., Gonthier P., Mellano M.G. (2020). Castanea spp. agrobiodiversity conservation: Genotype influence on chemical and sensorial traits of cultivars grown on the same clonal rootstock. Foods.

[66] Calani L., Bresciani L., Rodolfi M., Del Rio D., Petruccelli R., Faraloni C., Ganino T. (2022). Characterization of the (Poly) Phenolic Fraction of Fig Peel: Comparison among Twelve Cultivars Harvested in Tuscany. Plants.

[67] Torti S.D., Dearing M.D., Kursar T.A. (1995). Extraction of Phenolic Compounds from Fresh Leaves: A Comparison of Methods. J. Chem. Ecol..

[68] Petruccelli R., Ieri F., Ciaccheri L., Bonetti A. (2018). Polyphenolic Profiling and Chemometric Analysis of Leaves from Italian *Ficus carica* L. Varieties. Polyphenol Compounds in Common Fig. Eur. J. Hortic. Sci..

[69] Brand-Williams W., Cuvelier M.E., Berset C. (1995). Use of a Free Radical Method to Evaluate Antioxidant Activity. LWT-Food Sci. Technol..

[70] Cao G., Prior R.L. (1999). [5] Measurement of Oxygen Radical Absorbance Capacity in Biological Samples. Methods in Enzymology.

[71] Beghè D., Cirlini M., Beneventi E., Dall’Asta C., Marchioni I., Petruccelli R. (2024). Exploring Italian Autochthonous *Punica granatum* L. Accessions: Pomological, Physicochemical, and Aromatic Investigations. Plants.

[72] Weiss M.L. (1993). “DNA Fingerprinting: An Introduction”, by LT Kirby (Book Review). Hum. Biol..

[73] Brookfield J. (1996). A Simple New Method for Estimating Null Allele Frequency from Heterozygote Deficiency. Mol. Ecol..

[74] Botstein D., White R.L., Skolnick M., Davis R.W. (1980). Construction of a Genetic Linkage Map in Man Using Restriction Fragment Length Polymorphisms. Am. J. Hum. Genet..

[75] Marshall T., Slate J., Kruuk L., Pemberton J. (1998). Statistical Confidence for Likelihood-Based Paternity Inference in Natural Populations. Mol. Ecol..

[76] Kalinowski S.T., Taper M.L., Marshall T.C. (2007). Revising How the Computer Program CERVUS Accommodates Genotyping Error Increases Success in Paternity Assignment. Mol. Ecol..

[77] Anaconda Software Distribution (2020). Anaconda Doc. https://docs.anaconda.com/.

[78] Walt S., Van der Millman J. The Pandas development Pandas-Dev/Pandas: Pandas. Proceedings of the 9th Python in Science Conference.

[79] Virtanen P., Gommers R., Oliphant T.E., Haberland M., Reddy T., Cournapeau D., Burovski E., Peterson P., Weckesser W., Bright J. (2020). SciPy 1.0: Fundamental Algorithms for Scientific Computing in Python. Nat. Methods.

[80] Seabold S., Perktold J. Statsmodels: Econometric and Statistical Modeling with Python. Proceedings of the 9th Python in Science Conference.

[81] Vallat R. (2018). Pingouin: Statistics in Python. J. Open Source Softw..

[82] Waskom M. (2021). Seaborn: Statistical Data Visualization. J. Open Source Softw..

[83] Hunter J.D. (2007). Matplotlib: A 2D Graphics Environment. Comput. Sci. Eng..

[84] (2024). Lumivero LLC: XLSTAT Statistical and Data Analysis Solution. https://www.xlstat.com/en.

[85] Caruso T., Marra F., Costa F., Campisi G., Macaluso L., Marchese A. (2014). Genetic Diversity and Clonal Variation within the Main Sicilian Olive Cultivars Based on Morphological Traits and Microsatellite Markers. Sci. Hortic..

