# Supplementary material for: Description of Ficus carica L. Italian Cultivars II: Genetic and Chemical Analysis
Source: Plants (Basel). 2025 Apr 18;14(8):1238. doi: 10.3390/plants14081238 (PMC12030046; doi:10.3390/plants14081238)
Supplement: Supplementary file 1 [file plants-14-01238-s001.zip › plants-3536322-supplementary.pdf]

Table S1 Genetic profiles of 15 fig cultivars. Columns list the allele sizes for the 7 investigated loci (MFC, LMFC series)

CV	MFC2	MFC3	MFC4	LMFC12	LMFC24	LMFC30	LMFC31
PE	159/169	124/124	202/222	372/381	278/278	252/266	231/245
CO	167/173	128/128	202/226	375/401	276/276	260/262	231/245
SP	173/173	130/130	202/226	353/381	276/278	246/266	229/243
VE	173/173	132/138	222/222	353/381	276/280	246/262	231/245
PA	159/173	130/138	202/222	353/353	270/278	246/258	245/245
FI	159/173	126/138	202/226	353/353	278/278	258/266	245/245
DO	159/173	126/138	202/226	353/381	278/278	258/258	229/243
AL	159/173	126/138	202/226	353/381	278/278	260/260	231/245
BC	159/173	126/138	202/226	353/381	278/278	260/262	231/245
PO	173/179	126/130	202/226	353/353	276/278	258/258	245/245
GI	171/173	126/132	202/226	353/353	276/280	260/260	231/245
BB	159/159	130/130	222/222	353/381	278/278	236/262	231/131
PN	159/161	128/144	202/222	381/381	278/278	246/252	231/245
PB	161/173	132/136	222/226	381/381	276/276	246/252	231/245
BN	161/169	138/144	222/222	381/381	276/278	246/260	231/131

Table S2: Compounds'concentrations in leaves of 15 fig cultivars (mg/g DW). Relative content of hydroxycinnamic acids, flavonoid derivatives compounds and psoralen derivatives and their sums.

CV	Caffeic acid derivatives	p-coumaric acid derivatives	Apigenin derivatives	Quercetin derivatives	Kaempferol-derivatives	Psoralen derivatives	Total content
	**mg/g DW**	**mg/g DW**	**mg/g DW**	**mg/g DW**	**mg/g DW**	**mg/g DW**	**mg/g DW**
PA	6.801± 0.15e	1.352 ± 0.10c	0.651 ± 0.028ff	4.055 ± 0.16g	0.081 ± 0.00g-i	1.341 ± 0.030f	14.316 ± 0.29fg
AL	4.805 ± 0.26f	2.059±0.14b	0.792 ± 0.020de	4.120 ± 0.12g	0.096 ± 0.012gh	1.368 ± 0.038f	13.244 ± 0.33h
VE	7.947 ± 0.15c	4.032 ± 0.20a	0.898 ± 0.0.15cd	4.055 ± 0.26hi	0.154 ± 0.014e	1.503 ± 0.032h	18.073± 0.25f
PO	10.496±0.43a	0.826 ± 0.06de	0.473 ± 0.025g	3.326 ± 0.18gh	0.171 ± 0.016de	2.483 ± 0.039a	18.038 ± 0.25e
CO	6.338 ± 0.32e	2.195 ± 0.11b	0.880 ± 0.016d	5.474 ± 0.22f	0.195 ± 0.010cd	1.587 ± 0.033de	16.643 ± 0.18f
PN	3.810 ± 0.20g	1.034 ± 0.09c	0.283 ± 0.018h	5.079 ±0.19f	0.056 ± 0.00hi	1.715 ± 0.027c	12.2257 ± 0.27i
BB	4.274 ± 0.29fg	0.683 ± 0.08e	0.690 ± 0.011ef	1.825 ± 0.10j	0.111 ± 0.012fg	1.532 ± 0.038e	9.119±0.27j
GI	3.837 ± 0.18g	1.421 ± 0.12c	0.880 ± 0.023d	3.064 ± 0.27h	nd	nd	9.202 ± 022j
BC	9.266± 0.29b	1.471 ± 0.07c	1.183 ± 0.034b	12.995 ± 0.34a	0.455 ± 0.025a	1.684 ± 0.026cd	27.024± 0.01a
PE	10.334 ± 0.23a	1.340 ± 0.07c	0.869 ± 0.021d	9.543 ± 0.21bc	0.209 ± 0.016bc	1.326 ± 0.027f	23.62 ± 0.27b
PB	8.097 ± 0.17c	1.357 ± 0.06c	0.858 ± 0.017d	10.244 ± 0.36b	0.194 ± 0.011cd	1.122 ± 0.019g	21.884 ± 031c
BN	4.532 ± 0.23fg	2.210 ± 0.13b	1.431 ± 0.032a	8.584 ± 0.31d	0.243 ± 0.011c	2.167 ± 0.040b	19.162 ± 0.15d
DO	8.577 ± 0.19bc	0.848 ± 0.15de	.0.488 ± 0.012g	2.530 ± 0.18hi	0.069 ± 0.00hi	1.412 ± 0.022ef	13.928 ± 0.17gh
FI	7.130 ± 0.33d	1.239 ± 0.13c	0.997 ± 00276c	8.988 ± 0.25cd	0.335 ± 0.018b	1.393 ± 0.020ef	20.057 ± 0.29d
SP	6.464 ± 0.22d	1.163 ± 0.08cd	0.316 ± 0.017f	7.441 ± 0.26e	0.135 ± 0.011ef	0.359 ± 0.024i	16.683 ± 0.025f

Values are the means (*n*=3) ± SD. In each column different letters represent significant differences ($p < 0.05$) according to Tukey's HSD-test. AL, Albo; BB, Brogiotto Bianco; BC, Bianco di Carmignano; BN, Brogiotto Nero; CO, Corbo; DO, Dottato; FI, Fiorone; GI, Gigante di Carmignano; PA, Paradiso; PB, Pecciolo Bianco; PE, Perticone; PN, Pecciolo Nero; PO, Portogallo; SP, San Piero; VE, Verdino.

Table S3: Correlation coefficients between the main PCA components and the observed variables

Sucrose	0,911	0,242	-0,107	-0,207	0,156	-0,022	0,063	-0,072	0,008	-0,101	0,090	0,068	0,010
Glucose	-0,123	0,199	0,838	0,334	0,116	-0,116	0,237	-0,107	-0,157	0,003	0,087	-0,065	0,002
Fructose	0,909	0,034	-0,160	-0,272	-0,032	-0,032	0,001	-0,246	-0,060	0,043	-0,018	-0,066	-0,008
Total Sugars	0,935	0,133	-0,087	-0,233	0,051	-0,037	0,043	-0,192	-0,045	-0,014	0,031	-0,018	-0,001
Total Polyphenols	0,338	0,679	-0,127	0,007	-0,061	0,429	0,222	0,312	-0,270	0,035	-0,013	0,014	-0,002
DPPH EC50	-0,037	0,478	0,623	-0,267	0,077	0,411	-0,275	-0,051	0,179	-0,159	0,006	0,030	-0,005
ORAC	-0,473	-0,462	-0,201	-0,388	0,235	0,180	0,453	0,072	0,037	-0,262	0,025	-0,054	0,000
Flavonoids	0,031	-0,158	-0,236	0,687	0,354	0,453	0,160	-0,296	0,029	0,014	-0,038	0,032	-0,001
Caffeic acid derivatives	-0,305	0,554	0,081	-0,198	0,481	-0,442	0,332	-0,031	0,037	0,059	-0,091	0,057	-0,004
p-Coumaric acid derivatives	0,102	0,733	-0,163	0,208	-0,421	0,014	0,328	-0,006	0,306	0,023	-0,011	-0,050	0,002
Apigenin derivatives	-0,685	0,280	-0,220	0,108	-0,477	-0,182	0,053	-0,240	-0,156	-0,204	0,057	0,056	-0,004
Quercetin derivatives	-0,802	0,132	-0,200	-0,365	0,095	0,206	0,036	-0,106	0,039	0,272	0,166	0,006	-0,001
Kaempferol- derivatives	-0,710	0,489	-0,148	-0,286	0,075	0,140	-0,229	-0,202	-0,129	-0,015	-0,121	-0,055	0,008
Psoralen derivatives	-0,069	0,530	-0,436	0,360	0,433	-0,204	-0,348	0,137	0,026	-0,121	0,091	-0,052	-0,002
Eigenvalue	4,610	2,506	1,580	1,414	1,069	0,939	0,813	0,437	0,276	0,242	0,081	0,033	0,000
Variabily (%)	32,928	17,898	11,287	10,097	7,637	6,707	5,809	3,118	1,968	1,730	0,580	0,237	0,002
Cumulative Variance %	32,928	50,827	62,113	72,211	79,848	86,556	92,364	95,483	97,451	99,180	99,761	99,998	100,000
